# Association between endothelial activation and stress index and mortality in critically ill patients with atrial fibrillation: In MIMIC database: A Retrospective Cohort Study

**DOI:** 10.1371/journal.pone.0342664

**Published:** 2026-02-17

**Authors:** Peiling Zuo, Huanhuan Zhu, Chunying Sun, Xiaohan Ma, Sheng Chen, Rong Tang, Tong Wu, Ding Zhang, Xiao Tang, Wenquan Lv, Wenzhong Chen, Xiawei Wei, Encun Hou, Minsheng Wu, Minghe Jiang

**Affiliations:** 1 Graduate School, Guangxi University of Chinese Medicine, Nanning, Guangxi, China; 2 Ruikang Hospital Affiliated to Guangxi University of Chinese Medicine, Nanning, Guangxi, China; 3 Guangxi University of Chinese Medicine, Nanning, Guangxi, China; 4 Ruikang Hospital Affiliated to Guangxi University of Chinese Medicine Intensive Care Unit, Nanning, Guangxi, China; 5 Guangxi Hospital Division of The First Affiliated Hospital, Sun Yat-sen University, Nanning, Guangxi, China; Medical University of Vienna, AUSTRIA

## Abstract

**Background:**

Evidence indicates that the Endothelial Activation and Stress Index (EASIX) is a predictor of mortality in endothelium-related conditions; however, its association with mortality risk in atrial fibrillation (AF) remains uncertain. Accordingly, this study examines the relationship between EASIX and mortality risk among patients with AF.

**Methods:**

This retrospective analysis utilized data from the Medical Information Marketplace in Intensive Care IV (MIMIC-IV) database, which includes critically ill patients diagnosed with AF. To examine the association between EASIX scores and mortality, Kaplan–Meier survival analysis, Cox proportional hazards models, and restricted cubic spline regression were applied to evaluate the relationship between EASIX and all-cause mortality. Subgroup analyses were conducted to explore potential interactions with key patient characteristics, and sensitivity analyses were performed to further confirm the robustness of the results.

**Results:**

A total of 3,193 patients were included in the analysis. KM survival analysis showed that elevated EASIX levels were associated with a higher risk of both in-hospital and ICU mortality. After adjusting for potential confounders, increased EASIX levels remained significantly associated with in-hospital mortality [HR, 1.09 (95% CI 1.03, 1.15), P = 0.0002] and ICU mortality [HR, 1.10 (95% CI 1.04, 1.17), P = 0.0002]. Stratified analyses revealed a significant interaction between sepsis, respiratory failure, and EASIX in relation to both in-hospital and ICU mortality. To evaluate the robustness of the findings, a sensitivity analysis was performed. After additionally adjusting for metoprolol and heparin as covariates, patients in the highest EASIX group continued to demonstrate the greatest mortality risk: the HR for in-hospital death was 2.08 (95% CI: 1.51–2.85), and the HR for ICU death was 1.83 (95% CI: 1.21–2.65).

**Conclusion:**

Elevated EASIX levels correlate with higher mortality rates, underscoring its potential as an accessible tool for identifying high-risk patients and informing clinical decisions. However, further studies are needed to explore the underlying mechanisms and validate its applicability across diverse patient populations.

## 1. Introduction

Atrial fibrillation (AF) is the most common persistent arrhythmic disorder worldwide, placing a growing health burden on global populations [1]. It is characterized by severe disturbances in the electrical activity of the atria, impairing effective atrial contraction and leading to various complications and risks [2]. Currently, approximately 330 million people are affected by AF, with its prevalence rising significantly with age, particularly in those over 80 years [3]. Specifically, the prevalence of AF ranges from a relatively low 0.1% in individuals younger than 55 years old, to a notably higher 9.0% among those aged 80 and older [4]. Patients diagnosed with AF who are admitted to the Intensive Care Unit (ICU) often face a range of challenges, including multiple medical complications, complex health issues, and a significantly increased risk of death during their hospital stay [5]. Around 14% of ICU patients develop AF during hospitalization [6]. Despite its high prevalence among critically ill patients, there is a notable gap in research on reliable prognostic indicators for severe AF. Effectively identifying and managing these risk factors is crucial for reducing mortality in this high-risk population.

Endothelial activation and stress index (EASIX) is a composite score derived from routine laboratory parameters and is widely regarded as an effective tool for detecting endothelial injury. As research has progressed, EASIX has shown considerable promise for broader use across a range of clinical settings. However, unlike biomarkers such as Vascular Endothelial Growth Factor, EASIX does not directly measure endothelial dysfunction. Instead, it reflects a combination of factors that may be influenced by endothelial injury.

EASIX has been used to predict endothelial dysfunction and survival in patients after allogeneic cell transplantation and graft-versus-host disease [7,8], to predict sepsis before modulated treatment [9], and to forecast mortality in severe liver diseases [10]. The EASIX score is calculated using a simple formula that incorporates commonly measured laboratory parameters: serum lactate dehydrogenase (LDH) level (U/L), creatinine level (mg/dL), and platelet count (10^9/L) [7]. These accessible markers make EASIX practical for clinical use. Endothelial health plays a critical role in cardiovascular function, with conditions such as coronary heart disease, hypertension, neovascular disease, and AF closely linked to endothelial dysfunction [11]. The vascular endothelium is vital for maintaining vascular homeostasis, serving as a multifunctional organ responsible for regulating vascular tone. This regulation is essential for proper dilation and constriction of blood vessels, ensuring steady blood flow throughout the circulatory system, which is necessary for normal physiological processes and overall health [12]. Beyond regulating vascular tone, the endothelium also participates in crucial processes such as inflammation, coagulation, and immune responses.

Endothelial dysfunction is commonly found in patients with AF, and it is not just an incidental feature. Instead, it is strongly associated with more severe clinical outcomes, contributing to a significantly worse prognosis for those with AF [13]. The presence of endothelial dysfunction in these patients highlights its potential role in the progression of AF and underscores the need for targeted therapies aimed at restoring endothelial function. EASIX is an important indicator of endothelial dysfunction [14,15]. Previous research has highlighted the role of EASIX in evaluating endothelial health, emphasizing its potential to reflect the severity of endothelial injury or dysfunction, which is often associated with unfavorable cardiovascular outcomes. Building on these foundational findings, we propose that the EASIX index may not only serve as an essential diagnostic tool but could also be utilized as a robust prognostic marker in patients with AF. By forecasting potential outcomes and assisting in clinical decision-making, EASIX could facilitate more precise management of AF patients. Given the intricate nature of AF and the variability in patient responses to treatments, an effective tool like EASIX could significantly enhance individualized care. This approach would ensure that therapeutic interventions are tailored to meet the specific needs of each patient, potentially improving their overall treatment outcomes.

In this study, we aimed to explore the relationship between EASIX levels and mortality risk among AF patients by analyzing data from the Medical Information Mart for Intensive Care IV (MIMIC-IV), a comprehensive and well-established database of critically ill patients. This study demonstrates a robust and statistically significant association between elevated EASIX scores and a higher risk of all-cause mortality among patients with AF. Clinically, these findings suggest that EASIX may serve as a useful tool for clinicians to support prognostic assessment in individuals with AF. The early identification of individuals at high risk, through the application of EASIX, would enable clinicians to implement timely interventions, optimize patient monitoring, and personalize treatment plans, all of which could substantially improve the prognosis for those suffering from AF.

## 2. Materials

### 2.1. Data source

This retrospective analysis drew on clinical information from MIMIC-IV v3.0, an extensively used open-access repository for critical-care research. Curated by the MIT Laboratory for Computational Physiology, MIMIC-IV integrates de-identified, high-granularity records—spanning demographics, vital signs, laboratory measurements, procedures, and outcomes—from individuals managed in the ICUs at Beth Israel Deaconess Medical Center, a tertiary academic hospital recognized for state-of-the-art infrastructure and rigorous quality standards [16]. The comprehensive scope and high-quality data in this database make it an essential tool for researchers exploring a wide range of clinical questions. In accordance with MIMIC database usage protocols, one of the authors (Sheng Chen) completed a human subject research training course (ID: 66963781). Upon successful completion, Sheng Chen obtained credentialed user status on PhysioNet, required for accessing the MIMIC-IV database and using its data for research. Meanwhile the study was performed according to the guidelines of the Helsinki Declaration. The use of the MIMIC-IV database was approved by the review committee of Massachusetts Institute of Technology and Beth Israel Deaconess Medical Center. The data is publicly available (in the MIMIC-IV database), therefore, the ethical approval statement and the requirement for informed consent were waived for this study.

### 2.2. Study population

**Fig 1:**
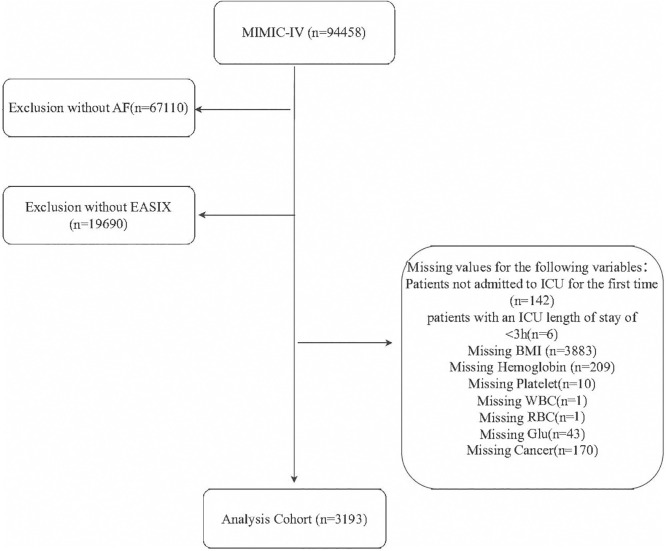
Flow of patients included in the trial.

In this study, patient data were retrieved from the MIMIC-IV database based on the following inclusion criteria. It is important to note that the MIMIC-IV database does not distinguish between paroxysmal and permanent AF. Therefore, all patients diagnosed with AF using International Classification of Diseases, Ninth Revision (ICD-9) diagnostic codes were included, encompassing both primary and secondary AF cases. The inclusion criteria were: (1) patients under 18 years of age at the time of their initial admission; (2) patients with multiple ICU admissions for AF, with only data from the initial admission included; (3) patients with severe comorbidities, including end-stage renal disease, cirrhosis, or cancer; (4) patients whose ICU stay was less than 3 hours; and (5) patients with incomplete biochemical data on the first day of admission. After applying these criteria, 3,193 patients were deemed eligible for the final analysis **(Fig 1)**. These patients were then divided into four groups based on the quartiles of EASIX scores, enabling a detailed investigation of outcomes at different risk levels. This structured approach allowed for a thorough analysis of the relationship between EASIX quartiles and clinical outcomes in this population.

### 2.3. Data collection

The study used R software for data extraction and organization, systematically categorizing the data into four key groups to ensure a comprehensive analysis. The first category included demographic information such as age, gender, race, and weight to characterize the basic patient profile. The second category focused on comorbidities, including conditions such as heart failure, respiratory failure, diabetes, paraplegia, and sepsis. This grouping reflects the prevalence of underlying conditions that may impact the patient’s prognosis.

The third category included laboratory markers measured within the first 24 hours of ICU admission, such as red blood cell (RBC) count, white blood cell (WBC) count, hemoglobin level, platelet count, serum sodium, serum creatinine, and glucose. These indicators provide valuable insights into the patient’s physiological and metabolic status, which are essential for assessing disease severity and prognosis.

The final category comprised disease severity scores recorded at the time of ICU admission, including the Glasgow Coma Scale (GCS) score, Acute Physiology Score III (APSIII), Simplified Acute Physiology Score II (SAPS-II), Oxford Acute Severity of Illness Score (OASIS), Sequential Organ Failure Assessment (SOFA), and Charlson Comorbidity Index (CCI). These scoring systems are commonly used in clinical practice to assess disease severity and predict prognosis, adding a critical dimension to the analysis [17,18].

Follow-up in this study began upon ICU admission and continued until the patient’s death, ensuring that the findings were closely linked to early variables collected within the first 24 hours of ICU stay. This structured approach provides a robust framework for exploring the relationships between baseline characteristics, laboratory findings, illness severity, and clinical outcomes.

### 2.4. Variable definitions

The EASIX score, a critical measure for assessing the severity of a patient’s condition, was calculated using the following formula: LDH level (U/L) multiplied by creatinine level (mg/dL), then divided by the platelet count (10^9/L). Previous research has shown that this score follows a skewed distribution, which can complicate statistical analysis. To address this, the EASIX score distribution was transformed using a base-2 logarithmic scale, improving the normality of the data and making it more suitable for statistical testing. The transformed EASIX scores were then categorized into quartiles, dividing the patient population into four distinct groups based on the score’s distribution [7].

Patients diagnosed with AF in the ICU were identified using ICD-9 and ICD-10 diagnostic codes, which provide a standardized method for classifying medical conditions. After excluding individuals without the necessary variables for analysis, the final cohort consisted of 3,193 patients. This cohort was carefully selected to ensure that only patients with complete and relevant data were included in the final analysis. All procedures adhered to the ethical guidelines outlined in the Declaration of Helsinki, ensuring that the research met the highest ethical standards.

### 2.5. Clinical outcomes

The main outcome of this study was all-cause mortality during hospitalization, while the secondary outcome focused on all-cause mortality specifically within the ICU.

### 2.6. Statistical analysis

In this study, critically ill patients diagnosed with AF were classified into four groups based on their EASIX score, which was the primary method for patient stratification. Baseline characteristics for each group were documented and analyzed to provide a comprehensive overview of demographic and clinical factors that could influence outcomes. Continuous variables were presented based on their distribution: as the mean ± standard deviation (SD) for normally distributed data, or as the median with interquartile range (IQR) for skewed data, ensuring accurate representation of central tendency and variability. Categorical variables were expressed as proportions, offering a clear distribution of categories within the study population.

The Kolmogorov-Smirnov test was used to assess the normality of the data. For normally distributed variables, either the t-test or analysis of variance (ANOVA) was employed to compare group means. For non-normally distributed data, the Mann-Whitney U test or Kruskal-Wallis test was used as robust alternatives to compare medians. Kaplan-Meier (KM) analysis was conducted to evaluate the incidence of various endpoints across EASIX score quartiles, with the log-rank test used to compare survival or event-free rates, providing insights into the potential impact of EASIX on prognosis. Hazard ratios (HR) and 95% confidence intervals (CI) were calculated using Cox proportional hazards models to evaluate the relative risk of events at different EASIX levels, adjusting for potential confounders. Statistical significance was set at a p-value of less than 0.05, ensuring the robustness of the results.

Multivariate models included the following: the crude model (unadjusted), model 1 (adjusted for sex, age, race, and weight, model 2 (further adjusted for CCI, GCS, OASIS, SAPS II, APS III, and SOFA), model 3 (additionally adjusted for sodium, glucose, serum creatinine, leukocytes, red blood cells, platelets, and hemoglobin), and model 4 (with the inclusion of TIA, stroke, sepsis, paraplegia, respiratory failure, and heart failure).

The first quartile of the EASIX score was selected as the reference value for the analysis, serving as the baseline for comparison across the other quartiles. This choice allows for a clear understanding of how higher or lower EASIX scores correlate with outcomes, relative to the lowest quartile. To assess potential trends, trend p-values were calculated across the quartiles, enabling a systematic evaluation of how the EASIX score varies in relation to key outcomes such as mortality. Additionally, the relationship between the EASIX score and in-hospital mortality was examined using restricted cubic splines, a statistical technique that allows for more flexible modeling of nonlinear relationships, thus providing a more accurate representation of how EASIX scores influence mortality risk.

To test the robustness of EASIX as a prognostic tool, we performed stratified analyses across subgroups defined by sex, age, and specific conditions (heart failure, respiratory failure, stroke, and sepsis). These analyses assessed whether the relationship between EASIX and mortality was consistent across subgroups. Interaction effects were evaluated using likelihood ratio tests. Additionally, sensitivity analyses were performed with further adjustment for metoprolol and heparin as covariates. Statistical significance was set at p < 0.05. All analyses were conducted in R.

## 3. Results

### 3.1. Baseline characteristics

A total of 3,193 AF patients were included in the final cohort. Differences in baseline characteristics between survivors and those who died during hospitalization are presented in **Table 1**. The mean age of the enrolled patients was 72.75 ± 12.31 years, with approximately 62.0% male and 67.8% white. The deceased group tended to be older (mean age 74 years) and had a higher prevalence of respiratory failure and sepsis. Disease severity scores were higher in the deceased group, while GCS scores were lower. Additionally, elevated WBC, serum creatinine, and glucose levels, along with lower hemoglobin and RBC counts, were observed in the deceased group (all P < 0.001). EASIX levels were significantly higher in the deceased group compared to the survivors (1.24 vs. 2.38, P < 0.0001).

**Table 1:** Baseline characteristics of the Survivors and Non-survivors.

Variable	Total (n = 3193)	Alive (n = 2398)	Death (n = 795)	P value
EASIX	1.49(0.42,2.79)	1.24(0.26,2.43)	2.38(1.12,3.70)	<0.0001
Sex				0.32
Female	1208(37.83)	895(37.32)	313(39.37)	
Male	1985(62.17)	1503(62.68)	482(60.63)	
Age	72.75 ± 12.31	72.24 ± 12.23	74.30 ± 12.43	<0.0001
Race				0.12
Black	268(8.39)	207(8.63)	61(7.67)	
Mexican American	1(0.03)	1(0.04)		
Other	756(23.68)	544(22.69)	212(26.67)	
White	2168(67.90)	1646(68.64)	522(65.66)	
Weight max	82.60(68.90,99.10)	83.00(69.40,99.20)	80.60(67.50,98.25)	0.31
Heart failure				<0.0001
yes	38(1.19)	17(0.71)	21(2.64)	
Respiratory failure				<0.0001
yes	1658(51.93)	1071(44.66)	587(73.84)	
Paraplegia				0.83
yes	7(0.22)	6(0.25)	1(0.13)	
Sepsis				<0.0001
yes	1113(34.86)	648(27.02)	465(58.49)	
Stroke				<0.01
yes	171(5.36)	113(4.71)	58(7.30)	
TIA				0.83
yes	7(0.22)	6(0.25)	1(0.13)	
Hemoglobin	10.37 ± 2.01	10.44 ± 2.00	10.16 ± 2.02	<0.001
Platelet	178.00(129.00,245.00)	179.00(132.00,245.00)	174.00(115.00,245.50)	0.18
RBC	3.50 ± 0.69	3.52 ± 0.69	3.42 ± 0.70	<0.001
WBC	12.00(8.30,17.20)	11.40(8.00,16.20)	14.40(9.80,21.00)	<0.0001
Serum creatinine	1.50(1.00,2.50)	1.30(0.90,2.30)	2.10(1.30,3.10)	<0.0001
Glu	139.00(111.00,184.00)	134.00(109.00,176.00)	154.00(120.00,208.00)	<0.0001
Sodium	138.77 ± 5.52	138.78 ± 5.26	138.75 ± 6.25	0.93
SOFA	7.00(4.00,10.00)	6.00(3.00,9.00)	9.00(6.00,12.00)	<0.0001
APSIII	54.00(40.00,71.00)	49.00(38.00,63.00)	71.00(55.00,88.50)	<0.0001
SAPSII	43.00(35.00,54.00)	40.00(33.00,50.00)	53.00(44.00,64.00)	<0.0001
OASIS	36.00(29.00,43.00)	34.00(28.00,40.00)	41.00(35.00,47.00)	<0.0001
GCS min	13.59 ± 2.90	13.71 ± 2.73	13.23 ± 3.32	<0.001
CCI	6.34 ± 2.52	6.18 ± 2.50	6.82 ± 2.55	<0.0001

Abbreviations: EASIX, endothelial activation and stress index; AF, Atrial fibrillation, BMI, Body mass index, RBC, red blood cell; WBC, white blood cell; GLU, glucose; SOFA, Sequential Organ Failure Assessment; CCI, Charlson comorbidity index; APSIII, Acute Physiology Score III, SAPS-II, Simplified Acute Physiology Score II, OASIS, Oxford Acute Severity of Illness Score; GCS, Glasgow Coma Scale; TIA, Transient ischemic attack.

Analysis revealed that different grouping methods produced similar results. Participants were divided into four quartiles based on EASIX, with baseline characteristics summarized in **Table 2**. Compared to Q1, patients in Q4 were younger, heavier, and had a higher proportion of males. From Q1 to Q4, WBC, serum creatinine, glucose, and EASIX showed an upward trend, while hemoglobin, platelets, RBCs, and sodium showed a downward trend. Severity scores (SOFA, APS III, SAPS II, OASIS, and CCI) generally increased with higher EASIX values. Moreover, the incidence of heart failure, respiratory failure, and sepsis increased from Q1 to Q4 (all P < 0.01). Compared with patients in Q1 (low EASIX), those in Q4 had longer ICU stays (3.77 days vs. 5.33 days, P < 0.0001), longer hospital stays (9.33 days vs. 11.96 days, P < 0.001), and higher in-hospital and ICU mortality rates (P < 0.0001). These findings suggest that higher EASIX values correlate with worse prognosis. No statistically significant differences were observed for paraplegia, stroke, or TIA (P > 0.05).

**Table 2:** Baseline characteristics and outcomes of participants classified by EASIX quartiles.

Variable	Total (n = 3193)	Q1 (n = 799)	Q2 (n = 795)	Q3 (n = 801)	Q4 (n = 798)	P value
Sex						<0.0001
Female	1208(37.83)	386(48.31)	306(38.49)	268(33.46)	248(31.08)	
Male	1985(62.17)	413(51.69)	489(61.51)	533(66.54)	550(68.92)	
Age	72.75 ± 12.31	72.98 ± 12.67	73.90 ± 12.22	73.64 ± 11.44	70.50 ± 12.62	<0.0001
Race						<0.0001
Black	268(8.39)	40(5.01)	62(7.80)	77(9.61)	89(11.15)	
Mexican American	1(0.03)	184(23.03)	186(23.40)	170(21.22)	216(27.07)	
Other	756(23.68)	575(71.96)	547(68.81)	553(69.04)	493(61.78)	
White	2168(67.90)			1(0.12)		
Weight max	82.60(68.90,99.10)	78.60(64.60,96.00)	81.00(67.95,97.65)	85.20(71.00,100.20)	85.60(71.50,100.98)	<0.0001
Heart failure						<0.001
yes	38(1.19)	2(0.25)	4(0.50)	13(1.62)	19(2.38)	
Respiratory failure						<0.0001
yes	1658(51.93)	355(44.43)	375(47.17)	425(53.06)	503(63.03)	
Paraplegia						0.17
yes	7(0.22)	4(0.50)	2(0.25)		1(0.13)	
Sepsis						<0.0001
yes	1113(34.86)	217(27.16)	220(27.67)	295(36.83)	381(47.74)	
Stroke						0.06
yes	171(5.36)	48(6.01)	48(6.04)	28(3.50)	47(5.89)	
TIA						0.67
yes	7(0.22)	1(0.13)	2(0.25)	3(0.37)	1(0.13)	
Hemoglobin	10.37 ± 2.01	10.41 ± 1.93	10.64 ± 1.97	10.29 ± 2.00	10.14 ± 2.10	<0.0001
Platelet	178.00(129.00,245.00)	249.00(188.50,330.50)	189.00(149.00,237.50)	158.00(123.00,213.00)	120.00(80.00,173.00)	<0.0001
RBC	3.50 ± 0.69	3.54 ± 0.64	3.59 ± 0.66	3.48 ± 0.70	3.39 ± 0.75	<0.0001
WBC	12.00(8.30,17.20)	11.80(8.20,16.80)	11.30(8.40,15.80)	12.00(8.40,17.10)	13.25(8.33,19.30)	<0.0001
Serum creatinine	1.50(1.00,2.50)	0.90(0.70,1.10)	1.30(1.00,1.70)	1.90(1.30,2.80)	2.90(2.00,4.50)	<0.0001
Glu	139.00(111.00,184.00)	125.00(106.00,159.00)	136.00(112.00,179.00)	142.00(115.00,190.00)	154.00(119.00,212.00)	<0.0001
Sodium	138.77 ± 5.52	138.85 ± 5.11	139.33 ± 4.99	138.62 ± 5.66	138.29 ± 6.18	<0.01
SOFA	7.00(4.00,10.00)	4.00(2.00,6.00)	5.00(3.00,8.00)	7.00(5.00,10.00)	10.00(8.00,13.00)	<0.0001
APSII	54.00(40.00,71.00)	44.00(34.00,55.50)	48.00(36.00,61.00)	56.00(44.00,71.00)	70.00(56.00,88.00)	<0.0001
SAPSII	43.00(35.00,54.00)	37.00(30.00,46.00)	39.00(33.00,48.50)	45.00(37.00,54.00)	54.00(44.00,63.00)	<0.0001
OASIS	36.00(29.00,43.00)	34.00(28.00,39.50)	34.00(28.00,40.50)	36.00(30.00,43.00)	39.00(33.00,47.00)	<0.0001
GCS min	13.59 ± 2.90	13.76 ± 2.62	13.73 ± 2.79	13.44 ± 3.08	13.45 ± 3.06	0.03
CCI	6.34 ± 2.52	5.39 ± 2.30	6.21 ± 2.41	6.90 ± 2.49	6.85 ± 2.59	<0.0001
LOS ICU, days	4.33(2.15,8.74)	3.77(1.96,7.47)	3.97(1.98,8.11)	4.55(2.38,8.93)	5.33(2.59,10.68)	<0.0001
LOS hospital, days	10.71(6.09,18.95)	9.33(5.94,16.98)	10.08(6.05,16.92)	11.63(6.74,19.38)	11.96(5.96,21.83)	<0.001
Outcome in hospital						<0.0001
alive	2398(75.10)	686(85.86)	661(83.14)	586(73.16)	465(58.27)	
	795(24.90)	113(14.14)	134(16.86)	215(26.84)	333(41.73)	
Outcome in ICU						<0.0001
alive	2563(80.27)	718(89.86)	702(88.30)	634(79.15)	509(63.78)	
death	630(19.73)	81(10.14)	93(11.70)	167(20.85)	289(36.22)	

Abbreviations: EASIX, endothelial activation and stress index; AF, Atrial fibrillation, BMI, Body mass index, RBC, red blood cell; WBC, white blood cell; GLU, glucose; SOFA, Sequential Organ Failure Assessment; CCI, Charlson comorbidity index; APSIII, Acute Physiology Score III, SAPS-II, Simplified Acute Physiology Score II, OASIS, Oxford Acute Severity of Illness Score; GCS, Glasgow Coma Scale; TIA, Transient ischemic attack.

### 3.2. EASIX and mortality

**Fig 2:**
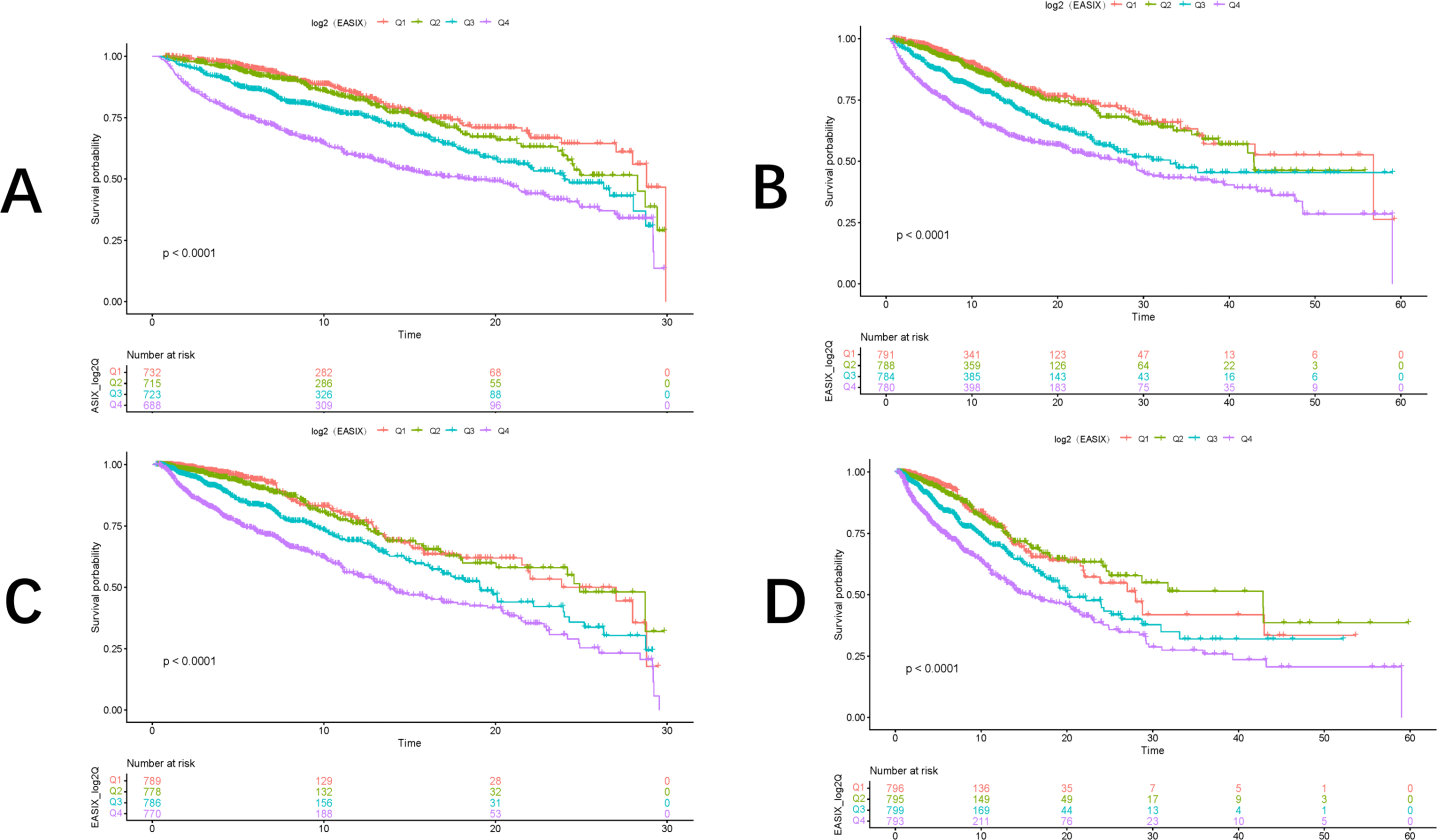
KM survival curves showing the risk of death in AF patients with varying EASIX values: (A) 30-day hospital mortality; (B) 60-day hospital mortality; (C) 30-day ICU mortality; (D) 60-day ICU mortality.

The incidence of the primary outcome across groups was analyzed using Kaplan-Meier survival curves based on EASIX quartiles. Patients with higher EASIX scores were found to have an increased risk of both in-hospital and ICU mortality, with the risk escalating as the length of hospitalization increased (P < 0.0001) (**Fig 2A–2D**).

To assess the relationship between the EASIX score and both in-hospital and ICU mortality in AF patients, four Cox proportional hazards regression models were developed **(Table 3).** The findings indicated that EASIX was a significant risk factor for both in-hospital and ICU mortality [HR, 1.09 (95% CI 1.03, 1.15), P = 0.0002; HR, 1.1 (95% CI 1.04, 1.17), P = 0.0002] when considered as a continuous variable.

**Table 3:** Cox regression analysis of EASIX and mortality in patients with AF.

character	Crude model		Model 1		Model 2		Model 3		Model 4	
	HR (95%CI)	P	HR (95%CI)	P	HR (95%CI)	P	HR (95%CI)	P	HR (95%CI)	P
Hospital mortality										
Continuous variable per unit	1.16(1.13,1.20)	<0.0001	1.2(1.16,1.24)	<0.0001	1.04(1.00,1.09)	0.05	1.08(1.03,1.14)	0.004	1.09(1.03,1.15)	0.002
Quartile										
Q1	ref		ref		ref		ref		ref	
Q2	1.14(0.89,1.47)	0.30	1.15(0.90,1.48)	0.27	1.03(0.80,1.33)	0.82	1.16(0.88,1.52)	0.29	1.17(0.89,1.53)	0.26
Q3	1.74(1.38,2.18)	<0.0001	1.83(1.45,2.30)	<0.0001	1.3(1.02,1.66)	0.03	1.58(1.20,2.09)	0.001	1.58(1.20,2.09)	0.001
Q4	2.57(2.08,3.19)	<0.0001	2.92(2.35,3.63)	<0.0001	1.48(1.15,1.90)	0.002	2.07(1.51,2.85)	<0.0001	2.07(1.50,2.84)	<0.0001
P for trend		<0.0001		<0.0001		<0.001		<0.0001		<0.0001
ICU mortality										
Continuous variable per unit	1.18(1.14,1.22)	<0.0001	1.22(1.17,1.26)	<0.0001	1.07(1.02,1.12)	0.01	1.1(1.03,1.16)	0.003	1.1(1.04,1.17)	0.002
Quartile										
Q1	ref		ref		ref		ref		ref	
Q2	1.08(0.80,1.45)	0.62	1.12(0.83,1.50)	0.47	1(0.74,1.36)	0.97	1.06(0.77,1.47)	0.70	1.06(0.77,1.46)	0.73
Q3	1.73(1.33,2.26)	<0.0001	1.82(1.39,2.37)	<0.0001	1.33(1.01,1.77)	0.04	1.49(1.08,2.06)	0.01	1.46(1.06,2.02)	0.02
Q4	2.57(2.01,3.29)	<0.0001	2.9(2.25,3.73)	<0.0001	1.5(1.12,2.00)	0.01	1.87(1.30,2.70)	<0.001	1.84(1.28,2.66)	0.001
P for trend		<0.0001		<0.0001		<0.001		<0.0001		<0.0001

Crude model: EASIX

Model1: EASIX, sex, age, race, weight max

Model2: EASIX, sex, age, race, weight max, CCI, GCS min, QASIS, SAPS-II, APSIII, SOFA

Model3: EASIX, sex, age, race, weight max, CCI, GCS min, QASIS, SAPS-II, APSIII, SOFA, Sodium, Glu, Serum creatinine, WBC, RBC, Platelet, Hemoglobin

Model4: EASIX, sex, age, race, weight max, CCI, GCS min, QASIS, SAPS-II, APSIII, SOFA, Sodium, Glu, Serum creatinine, WBC, RBC, Platelet, Hemoglobin, TIA, Stroke, Sepsis, Paraplegia, Respiratory failure, Heart failure

Abbreviations: EASIX, endothelial activation and stress index; AF, Atrial fibrillation, BMI, Body mass index, RBC, red blood cell; WBC, white blood cell; GLU, glucose; SOFA, Sequential Organ Failure Assessment; CCI, Charlson comorbidity index; APSIII, Acute Physiology Score III, SAPS-II, Simplified Acute Physiology Score II, OASIS, Oxford Acute Severity of Illness Score; GCS, Glasgow Coma Scale; TIA, Transient ischemic attack.

When EASIX was stratified, patients in the higher quartiles showed a significant association with an increased risk of in-hospital mortality across all four Cox proportional hazards models [HR, 2.07 (95% CI 1.50, 2.84), P < 0.0001]. The risk of death progressively increased with higher EASIX values, with patients in the highest quartile exhibiting a markedly higher risk of in-hospital death compared to those in the lowest quartile. These findings were consistent in a multivariate Cox proportional hazards analysis for ICU mortality.

**Fig 3:**
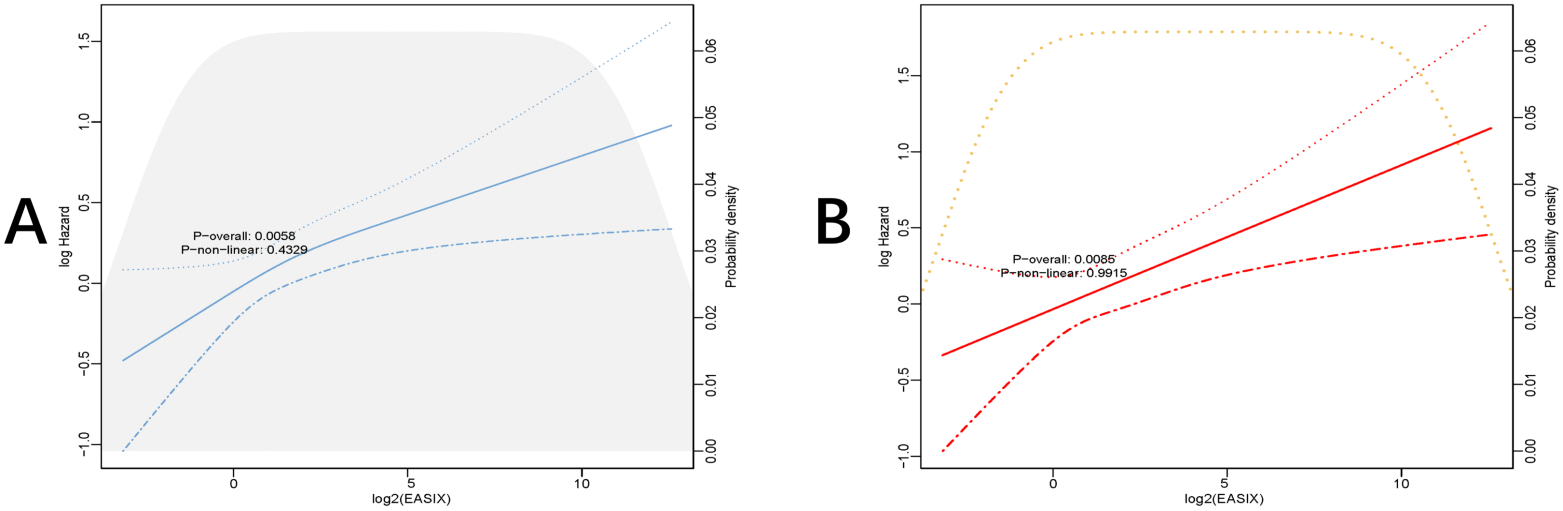
Restricted cubic spline curves illustrating the EASIX hazard ratio. **(A)** Restricted cubic spline for in-hospital mortality. **(B)** Restricted cubic spline for ICU mortality.

Additionally, restricted cubic spline regression analyses revealed a linear relationship between increasing EASIX levels and both in-hospital and ICU mortality risks (P-overall: 0.0058, P-non-linear: 0.4329; P-overall: 0.0085, P-non-linear: 0.9915) **(Fig 3A**,**3B).**

### 3.3. Subgroup analysis

**Fig 4:**
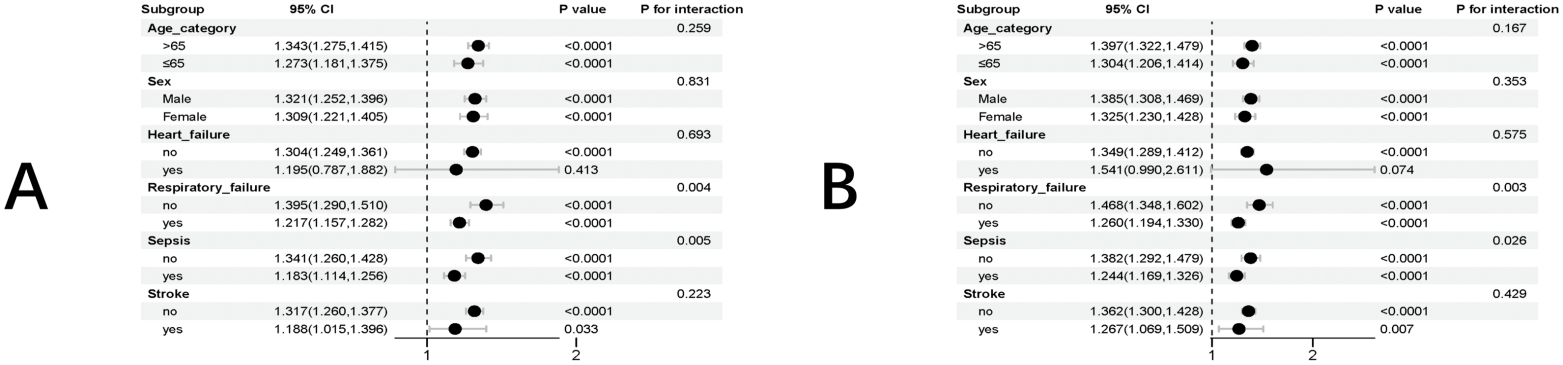
Forest plots of hazard ratios in different subgroups. **(A)** Forest plots of hazard ratios for the hospital mortality in different subgroups. **(B)** Forest plots of hazard ratios for the ICU mortality in different subgroups.

Subgroup analyses were conducted to assess the validity of EASIX in stratifying the risk of death based on various factors, including age, sex, BMI, heart failure, respiratory failure, sepsis, and stroke. In the stratified analysis of in-hospital mortality, a significant interaction was found between respiratory failure, sepsis, and EASIX (P for interaction = 0.004, P for interaction = 0.005). EASIX was significantly associated with the risk of in-hospital mortality in the following subgroups of patients with AF: age > 65 or ≤65, male, female, without heart failure, with or without respiratory failure, with or without sepsis, and with or without stroke (all P < 0.05) **(Fig 4A)**

Similarly, in the stratified analysis of ICU mortality, a significant interaction was observed between sepsis, respiratory failure, and EASIX (P for interaction = 0.026, P for interaction = 0.003) **(Fig 4B)**. Across all subgroups, EASIX was significantly associated with a higher risk of ICU mortality in the following groups: age > 65 or ≤65, male, female, without heart failure, with or without respiratory failure, with or without sepsis, and without stroke (all P < 0.05).

### 3.4. Sensitivity analyses

In the sensitivity analysis, after further adjustments for metoprolol and heparin as covariates, the HR for EASIX remained robust (S1 Table). Notably, when AF patients were stratified by EASIX quartiles, individuals in the highest quartile (Q4) consistently exhibited the highest risk. For in-hospital mortality, the HR for Q4 was 2.08 (95% CI: 1.51–2.85), and for ICU mortality, it was 1.83 (95% CI: 1.21–2.65), both showing statistically significant associations.

## 4. Discussion

Our study thoroughly examined the relationship between EASIX and both hospital and ICU mortality in AF patients using the MIMIC database. We found that elevated EASIX levels were associated with increased mortality in both hospital and ICU settings, even after adjusting for potential confounders. The observed linear dose-response relationship suggests that in inpatient and intensive care environments, AF patients with higher EASIX levels, longer hospital or ICU stays, and higher mortality rates are at greater risk of death. This highlights the clinical value of early risk stratification. As such, EASIX may serve as an effective decision-support tool for clinicians and could potentially act as an independent risk factor for critically ill AF patients.

In contrast to other arrhythmias, AF is the most common type, with its prevalence rising significantly as individuals age [19]. The likelihood of developing AF increases with age, and this trend becomes more pronounced with each passing decade, making AF a major concern in geriatric medicine. Additionally, AF extends beyond the atria, affecting multiple systems within the body. It is a multifaceted disorder intricately linked to various physiological dysfunctions, including systemic inflammation, endothelial dysfunction, and metabolic disorders. AF is also associated with significant changes in myocardial structure and function, further complicating its diagnosis and management [20]. Despite extensive research, the full pathophysiological mechanisms underlying AF remain incompletely understood, with many aspects still under active investigation. A substantial body of experimental and clinical studies has shown that AF is closely associated with dysfunction in both systemic vascular and atrial endothelial cells. This relationship is mediated by several interconnected and complex mechanisms that contribute to disease progression. These mechanisms include: (1) alterations in hemodynamics affecting blood flow, (2) increased shear stress on endothelial cells, (3) reduced bioavailability of NO, (4) elevated oxidative stress and inflammation throughout the vascular system, (5) disturbances in the renin-angiotensin axis that affect vascular tone, and (6) intracellular calcium (Ca2+) overload that disrupts cellular function [21–23].

First, shear stress is specifically induced by increased blood flow within the vasculature. This flow-mediated shear stress plays a pivotal role in regulating the expression of nitric oxide (NO) synthase, with a notable downregulation of nitric oxide synthase (NOS) occurring at low-flow sites, which affects endothelial function [24–26]. Since AF results in the loss of organized atrial contraction, it predisposes patients to decreased left atrial blood flow. As a result, AF is associated with a significant reduction in the expression of endothelial NO synthase and a subsequent decline in NO bioavailability [21]. The reduced availability of NO, along with the impairment of endothelial function, likely explains the pathophysiological mechanisms through which AF contributes to endothelial dysfunction.

Dimethylarginine, including asymmetric (ADMA) and symmetric (SDMA) forms, contributes to endothelial dysfunction in AF. Both are methylated derivatives of L-arginine, the precursor of NO essential for endothelial health. Elevated ADMA inhibits NOS, leading to oxidative stress and inflammation [27,28], while SDMA reduces L-arginine uptake, limiting NO synthesis [29]. Increased ADMA and SDMA levels in AF patients suggest their role in endothelial impairment [30–33].

In addition to the effects of dimethylarginine, AF itself induces atrial inflammation, leading to elevated levels of C-reactive proteins and cytokines. These inflammatory markers exert pro-inflammatory effects on endothelial cells, which may further exacerbate endothelial dysfunction and contribute to the progression of AF [22]. Simultaneously, the renin-angiotensin system plays a pivotal role in this process, as angiotensin II has been shown to promote atrial cell death, creating a vicious cycle that perpetuates AF. Improved endothelial function has been recognized as an important clinical marker of altered atherogenic risk factors, underscoring the importance of endothelial health in managing AF and its associated cardiovascular risks [34].

Although EASIX is not a biomarker, recent studies have consistently shown a robust and well-established link between endothelial function and both the onset and progression of AF [35,36]. This growing body of evidence emphasizes the critical role of endothelial health in the pathophysiology of AF, suggesting that endothelial dysfunction may significantly contribute to the initiation and exacerbation of this arrhythmia. Vascular endothelial cell dysfunction is increasingly proposed as a key cause of the thrombotic state commonly associated with AF, further complicating the clinical picture for affected patients [37]. One plausible explanation is that endothelial cell dysfunction reduces the bioavailability of NO, which is essential for maintaining proper vascular function. This reduction impairs the ability of NO to inhibit thrombosis and promote healthy blood flow, potentially contributing to the heightened thrombotic risk observed in AF patients [38].

Another key factor in this process is von Willebrand factor (vWF), a glycoprotein synthesized by endothelial cells in response to endothelial injury. Elevated vWF levels affect platelet function [39], promoting platelet adhesion, aggregation, and thrombus formation, which increases thrombosis risk in AF patients and leads to complications such as stroke and systemic embolism [40]. High vWF levels are recognized as markers of endothelial dysfunction and serve as useful biomarkers for assessing endothelial damage in AF [41]. They also contribute to oxidative stress, inflammation, and atherosclerosis, further worsening endothelial dysfunction and promoting AF progression [42]. However, it is important to note that, unlike biomarkers such as Vascular Endothelial Growth Factor, EASIX does not directly measure endothelial dysfunction but rather reflects a combination of factors that may be influenced by endothelial injury.

This study demonstrates a strong, statistically significant association between elevated EASIX scores and increased all-cause mortality in AF patients. Clinically, this suggests that EASIX is a valuable prognostic tool, objectively identifying patients at higher risk of poor outcomes and supporting timely, informed decision-making. As it can be easily calculated from routine hospitalization data, EASIX provides a practical, real-time method for risk assessment. Incorporating EASIX into clinical practice enables early risk stratification, allowing clinicians to prioritize care, implement targeted interventions, and personalize treatment. Ultimately, its use can enhance AF management by improving prognosis-based care and optimizing clinical outcomes and resource allocation. Although our study indicates a strong association between elevated EASIX levels and poor outcomes, including in-hospital and ICU mortality, causality cannot be inferred from these observational findings. Further prospective and mechanistic studies are needed to explore the potential role of EASIX in predicting outcomes.

One of the main strengths of this study is that it represents the first investigation into the relationship between EASIX and prognosis in patients with AF, providing valuable new insights into its clinical utility. Additionally, the study benefits from the extensive and diverse population data available in the MIMIC-IV database, allowing for rigorous statistical adjustments to account for potential confounders. However, the design of this study as a single-center cohort introduces several limitations. Single-center studies are more prone to selection bias, which may limit the generalizability of the findings to broader patient populations. Specifically, the MIMIC database originates from an academic medical center in the United States, where patient demographics, clinical practices, and healthcare systems may differ from those in other countries or regions. As a result, our findings may not be directly applicable to ICU populations outside the United States. Furthermore, the MIMIC-IV database poses challenges in obtaining specific clinical features for atrial fibrillation, such as the lack of detailed treatment data and data related to electrical or pharmacological cardiac rhythm reversal. This restriction may limit the depth of our analysis and introduce potential confounding factors that could affect the interpretation of the results. Other limitations of this study include residual confounding, lack of treatment data, and potential coding bias. While we adjusted for multiple confounding factors such as demographic characteristics, comorbidities, and laboratory markers, residual confounding may still affect the accuracy of the results due to the absence of detailed treatment information (e.g., anticoagulation therapy, heart rate control). The lack of treatment data in the MIMIC-IV database also limits our ability to comprehensively assess the prognosis of patients.

Future research can reduce bias and enhance the broad applicability of the results in several ways. First, multi-center studies involving patients from different regions should be conducted to improve the external validity of the findings. Second, a prospective cohort design should be adopted to collect real-time treatment data, such as anticoagulation therapy and heart rate control, in order to reduce the bias associated with retrospective data. Additionally, future research should gather more detailed treatment information, particularly regarding the specific clinical features of AF (e.g., persistent or paroxysmal atrial fibrillation), to more comprehensively assess the relationship between EASIX and treatment.

To minimize residual confounding bias, advanced statistical methods, such as propensity score matching or instrumental variable analysis, can be employed in future studies. Expanding the racial and regional diversity of the research population, especially verifying the applicability of EASIX among non-Western populations, will also enhance its global applicability. Finally, the use of ICD-9 diagnostic codes in this study may introduce coding biases, potentially leading to inaccurate disease classification and outcome determination. To address these limitations, future research should adopt prospective data collection, improved coding methods, and more detailed treatment information to further verify the application of EASIX in various clinical settings.

Despite these limitations, the study makes a significant contribution to AF research, highlighting the potential of EASIX as a valuable prognostic marker. Future research should aim to address these limitations by incorporating more comprehensive AF characterization metrics and including a wider range of clinical settings, thereby better elucidating the role of EASIX in predicting patient outcomes in AF.

## 5. Conclusion

Elevated EASIX levels in patients with AF were significantly associated with increased in-hospital and ICU mortality, highlighting its potential as a prognostic marker. Clinicians could use EASIX to identify AF patients at higher risk, enabling better patient stratification and more targeted interventions.

## Supporting information

S1 Table Sensitivity Analysis: Cox regression analysis of EASIX and mortality in AF patients after additional adjustment for Metoprolol and Heparin as covariates. (DOCX)

## Abbreviations

EASIX: Endothelial activation and stress index
AF: Atrial fibrillation
MIMIC-IV: Medical Information Marketplace in Intensive Care IV
ICU: Intensive Care Unit
KM: Kaplan-Meier
BMI: Body mass index
RBC: Red blood cell
WBC: White blood cell
GLU: Glucose
APSIII: Acute Physiology Score III
SAPS-II: Simplified Acute Physiology Score II
OASIS: Oxford Acute Severity of Illness Score
SOFA: Sequential Organ Failure Assessment
OASIS: Oxford Acute Severity of Illness Score
GCS: Glasgow Coma Scale
TIA: Transient ischemic attack
HRs: Hazard ratios
Cis: Confidence intervals
ADMA: Asymmetric dimethylarginine
SDMA: Symmetric dimethylarginine
NO: Nitric oxide
NOS: Nitric oxide synthase
vWF: von Willebrand factor
