# Supplementary material for: Association between endothelial activation and stress index and mortality in critically ill patients with atrial fibrillation: In MIMIC database: A Retrospective Cohort Study
Source: PLoS One. 2026 Feb 17;21(2):e0342664. doi: 10.1371/journal.pone.0342664 (PMC12912572; doi:10.1371/journal.pone.0342664)
Supplement: S1 Table — (DOCX) [file pone.0342664.s001.docx]

**S1 Table**. Sensitivity Analysis: Cox regression analysis of EASIX and mortality in AF patients after additional adjustment for Metoprolol and Heparin as covariates.

| character | Crude model | | Model 1 | | Model 2 | | Model 3 | | Model 4 | |
| --- | --- | --- | --- | --- | --- | --- | --- | --- | --- | --- |
|  | HR (95%CI) | P | HR (95%CI) | P | HR (95%CI) | P | HR (95%CI) | P | HR (95%CI) | P |
| Hospital mortality |  |  |  |  |  |  |  |  |  |  |
| Continuous variable per unit | 1.16(1.13,1.20) | <0.0001 | 1.2(1.16,1.24) | <0.0001 | 1.04(1.00,1.09) | 0.05 | 1.08(1.03,1.14) | 0.004 | 1.09(1.03,1.15) | 0.002 |
| Quartile |  |  |  |  |  |  |  |  |  |  |
| Q1 | ref |  | ref |  | ref |  | ref |  | ref |  |
| Q2 | 1.14(0.89,1.47) | 0.30 | 1.15(0.90,1.48) | 0.27 | 1.03(0.80,1.33) | 0.82 | 1.16(0.88,1.52) | 0.29 | 1.18(0.81,1.54) | 0.24 |
| Q3 | 1.74(1.38,2.18) | <0.0001 | 1.83(1.45,2.30) | <0.0001 | 1.3(1.02,1.66) | 0.03 | 1.58(1.20,2.09) | 0.001 | 1.56(1.21,2.09) | 0.001 |
| Q4 | 2.57(2.08,3.19) | <0.0001 | 2.92(2.35,3.63) | <0.0001 | 1.48(1.15,1.90) | 0.002 | 2.07(1.51,2.85) | <0.0001 | 2.08(1.51,2.85) | <0.0001 |
| P for trend |  | <0.0001 |  | <0.0001 |  | <0.001 |  | <0.0001 |  | <0.0001 |
| ICU mortality |  | |  | |  | |  | |  | |
| Continuous variable per unit | 1.18(1.14,1.22) | <0.0001 | 1.22(1.17,1.26) | <0.0001 | 1.07(1.02,1.12) | 0.01 | 1.1(1.03,1.16) | 0.003 | 1.1(1.04,1.17) | 0.002 |
| Quartile |  |  |  |  |  |  |  |  |  |  |
| Q1 | ref |  | ref |  | ref |  | ref |  | ref |  |
| Q2 | 1.08(0.80,1.45) | 0.62 | 1.12(0.83,1.50) | 0.47 | 1(0.74,1.36) | 0.97 | 1.06(0.77,1.47) | 0.70 | 1.06(0.77,1.46) | 0.73 |
| Q3 | 1.73(1.33,2.26) | <0.0001 | 1.82(1.39,2.37) | <0.0001 | 1.33(1.01,1.77) | 0.04 | 1.49(1.08,2.06) | 0.01 | 1.47(1.07,2.03) | 0.25 |
| Q4 | 2.57(2.01,3.29) | <0.0001 | 2.9(2.25,3.73) | <0.0001 | 1.5(1.12,2.00) | 0.01 | 1.87(1.30,2.70) | <0.001 | 1.83(1.21,2.65) | 0.001 |
| P for trend |  | <0.0001 |  | <0.0001 |  | <0.001 |  | <0.0001 |  | <0.0001 |

Crude model: EASIX_log2

Model1: EASIX_log2, sex, age, race, weight max

Model2: EASIX_log2, sex, age, race, weight max, CCI, GCS min, QASIS, SAPS-II, APSIII, SOFA

Model3: EASIX_log2, sex, age, race, weight max, CCI, GCS min, QASIS, SAPS-II, APSIII, SOFA, Sodium, Glu, Serum creatinine, WBC, RBC, Platelet, Hemoglobin

Model4: EASIX_log2, sex, age, race, weight max, CCI, GCS min, QASIS, SAPS-II, APSIII, SOFA, Sodium, Glu, Serum creatinine, WBC, RBC, Platelet, Hemoglobin, TIA, Stroke, Sepsis, Paraplegia, Respiratory failure, Heart failure, Metoprolol, Heparin

Abbreviations: EASIX, endothelial activation and stress index; AF, Atrial fibrillation, BMI, Body mass index, RBC, red blood cell; WBC, white blood cell; GLU, glucose; SOFA, Sequential Organ Failure Assessment; CCI, Charlson comorbidity index; APSIII, Acute Physiology Score III, SAPS-II, Simplified Acute Physiology Score II, OASIS, Oxford Acute Severity of Illness Score; GCS, Glasgow Coma Scale; TIA, Transient ischemic attack.
